# Towards Monitoring Biodiversity in Amazonian Forests: How Regular Samples Capture Meso-Scale Altitudinal Variation in 25 km^2^ Plots

**DOI:** 10.1371/journal.pone.0106150

**Published:** 2014-08-29

**Authors:** Darren Norris, Marie-Josée Fortin, William E. Magnusson

**Affiliations:** 1 Programa de Pós-Graduação em Biodiversidade Tropical, Universidade Federal do Amapá, Macapá, Amapá, Brazil; 2 Department of Ecology & Evolutionary Biology, University of Toronto, Ontario, Canada; 3 Coordenação de Biodiversidade, Instituto Nacional de Pesquisas da Amazônia, Manaus, Amazonas, Brazil; Cirad, France

## Abstract

**Background:**

Ecological monitoring and sampling optima are context and location specific. Novel applications (e.g. biodiversity monitoring for environmental service payments) call for renewed efforts to establish reliable and robust monitoring in biodiversity rich areas. As there is little information on the distribution of biodiversity across the Amazon basin, we used altitude as a proxy for biological variables to test whether meso-scale variation can be adequately represented by different sample sizes in a standardized, regular-coverage sampling arrangement.

**Methodology/Principal Findings:**

We used Shuttle-Radar-Topography-Mission digital elevation values to evaluate if the regular sampling arrangement in standard RAPELD (rapid assessments (“RAP”) over the long-term (LTER [“PELD” in Portuguese])) grids captured patters in meso-scale spatial variation. The adequacy of different sample sizes (*n* = 4 to 120) were examined within 32,325 km^2^/3,232,500 ha (1293×25 km^2^ sample areas) distributed across the legal Brazilian Amazon. Kolmogorov-Smirnov-tests, correlation and root-mean-square-error were used to measure sample representativeness, similarity and accuracy respectively. Trends and thresholds of these responses in relation to sample size and standard-deviation were modeled using Generalized-Additive-Models and conditional-inference-trees respectively. We found that a regular arrangement of 30 samples captured the distribution of altitude values within these areas. Sample size was more important than sample standard deviation for representativeness and similarity. In contrast, accuracy was more strongly influenced by sample standard deviation. Additionally, analysis of spatially interpolated data showed that spatial patterns in altitude were also recovered within areas using a regular arrangement of 30 samples.

**Conclusions/Significance:**

Our findings show that the logistically feasible sample used in the RAPELD system successfully recovers meso-scale altitudinal patterns. This suggests that the sample size and regular arrangement may also be generally appropriate for quantifying spatial patterns in biodiversity at similar scales across at least 90% (≈5 million km^2^) of the Brazilian Amazon.

## Introduction

Gathering sample data that reliably reflects the underlying spatial and temporal variability in target measurements is a fundamental requisite for ecological studies. Bias (sampling bias, estimation bias, etc.) occurs when the sampling design used induces errors and artificial differences in the values among samples [1], [2]. These first principles form the basis of many an undergraduate statistics course but beyond lecture halls obtaining reliable and representative sample data is one of the primary challenges for any biodiversity monitoring program [3], [4], where the necessary requirement is that it is sensible and meaningful to compare and contrast sample values [1], [3], [4].

There is no panacea in ecological sampling because sampling optima are context and location specific. Novel applications (e.g., monitoring of biodiversity for payments of environmental services) present further challenges and call for renewed efforts to establish general monitoring guidelines and frameworks, especially in biodiversity rich areas [5], [6], [7]. A particular challenge to establishing payments for biodiversity services is that reliable and robust long-term monitoring of biodiversity indicators is required to ensure that services are and will continue to be provided. Such long-term monitoring of biodiversity requires decisions today about the sampling distribution to recover information about changes in response to future, and often unknown, threats.

While the adequacy of sampling for a specific threat might not be known, we do know that most analyses require knowledge of the spatial structure, and hence the distribution patterns of response variables. If the distribution of sampling is inadequate to capture the spatial patterns in biodiversity variables, any subsequent analyses are likely to be inefficient and/or biased. Design of an optimal spatial sampling scheme requires a careful balance between sampling locations that are too close to one another, thus not providing enough new information (highly autocorrelated data), and sampling locations that are too sparse, so that processes at other spatial scales introduce too much variability [8]. Within biodiversity monitoring programs, sample quality (representativeness) and sample detail (resolution) often require separate investment in time, money and expertise (p251 of [4]). Finite resources mean that any biodiversity monitoring program faces a tension between securing samples that are of both sufficient quantity (to ensure that results can be extrapolated across scales that are relevant to management) and quality (to ensure they reliably capture the underlying variability in the measurement of interest) for addressing the research objective at hand [3], [4].

Field (on-the-ground) data collection provides the empirical basis upon which our understanding of biodiversity conservation is built. When a non-contiguous “sparse” [9] sampling strategy is used (be it regular, stratified, random or clustered), the extent is not completely surveyed and information is missing about the spatial pattern [9]. Sample pattern (geometrical configuration of the sample elements in space) and sample density (number of samples per unit area) are recognized as two important determinants for optimizing sparse sampling designs [10]. However, there is no information on the distribution of most biodiversity variables across the Amazon basin with the precision necessary to test the adequacy of sampling density and configuration. Faced with such uncertainty, ecologists often turn to rules of thumb, such as a minimum of 30 sample locations are required to detect significant spatial autocorrelation [9], and the reliable estimation of spatial structure and spatial model parameters may require 100 or more sampling locations [9]. If other factors remain unchanged, sparse sample patterns ranked by decreasing level of logistical efficiency are regular, stratified, random, and clustered [10].

Advances in statistical methodologies and computational power mean that there are a wide variety of approaches that can be applied to deal with sampling designs that were traditionally considered as suboptimal/inappropriate due to lack of spatial independence between samples [11]. However, for the statistics to be meaningful and useful, it is vital to understand how well the sampling captures the variation in the target variable. Although the substantial body of geostatistical literature enables us to generate well informed expectations of how well a sample captures patterns in known abiotic and biotic attributes, it is hard to establish informed expectations/guidelines when there is little or no knowledge regarding the response of interest [12].

Although there is little information on the distribution of most biodiversity variables across the Amazon basin, altitude is a driver and modulator of species distribution patterns from microhabitat to biogeographic scales [13], [14], [15], [16]. Altitude not only affects soil, water availability, climate and a myriad of other abiotic and biotic variables [16], it is also a key determinant of Amazonian biodiversity [17], [18], [19], [20], [21]. As such, spatial variation in altitude and biodiversity are expected to be strongly correlated, especially at local (10 m–1 km [19]) to meso-scales (1 km–100 km [13]).

We can identify at least three reasons why altitude is also perhaps the only variable available that can be used to provide a baseline evaluation of sample arrangements for biodiversity monitoring. Firstly, freely available digital elevation models (such as those obtained from ASTER/SRTM satellite images) provide a continuous global coverage of known altitude values at resolutions <100 m (resolutions vary slightly with distance from the equator). Secondly, not only is the variation in many response variables associated with the variation in altitude, but in the majority of situations, it will also be necessary to model and/or control for the effects of altitude in both within and between site comparisons. For example, altitude is used to predict soil characteristics and both can then be modeled as covariates for numerous response variables [22], [23], [24], [25], [26]. Thirdly, on the scales relevant to biodiversity monitoring (i.e. excluding changes that occur on geological timescales) meso-scale altitude values are dependent simply on the geographical location. Therefore it should be possible to accurately predict altitude using only spatial predictors, i.e. by modeling geographic coordinates. These three reasons make altitude a useful metric for evaluating the adequacy of sampling arrangements to represent meso-scale spatial variation.

Altitude can be particularly informative for evaluating sample arrangements for biodiversity monitoring in cases (i) when strata are not identifiable; (ii) there is uncertainty in the expected changes in the spatial arrangement of responses [12] (i.e. biodiversity indicator variables) to future, and often unknown, threats and/or (iii) where questions involve investigating responses of multiple indicators [27], [28]. Here, we use data from the most widespread system of standardized biodiversity monitoring in the Amazon (RAPELD [3], [22], [29]) to investigate whether regular sampling at 1-km intervals is sufficient to accurately recover spatial patterns in altitude within 25 km^2^ sampling grids across the Brazilian Amazon. To examine the accuracy of both samples and associated predictions within the survey area we focus on both sample and interpolated values within grids. Specifically we address the following questions:

1. How do sample size and sample standard deviation influence the representativeness of values from regularly arranged samples and the associated spatially interpolated altitude values?
2. How does sample size and sample standard deviation influence the similarity and accuracy of spatially interpolated altitude values?
3. What are the lower thresholds in sample size and sample standard deviation needed to obtain representative, similar and accurate values?
4. What are the practical implications of these findings for the use of regular meso-scale sample arrangements in Amazon forest biodiversity monitoring programs?

## Materials and Methods

### Study areas and sample arrangements

**Figure 1:**
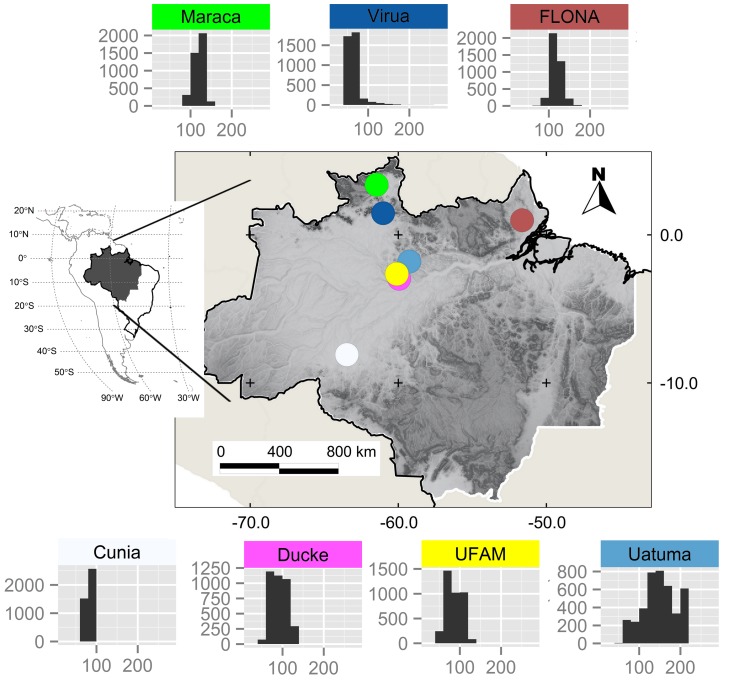
Distribution of seven active research sites within the Brazilian Amazon. Site locations (circles) are overlaid on a 30 arc-second shaded relief map derived from mean altitude values [45] distributed by the U.S. Geological Survey (USGS) Earth Resources Observation & Science (EROS) Center (http://topotools.cr.usgs.gov/gmted_viewer/). Histograms show the frequency distribution of SRTM altitude values (90 m resolution) within the study sites, with counts (y-axis) grouped in 20 meter altitude bins (x-axis).

To investigate how well a regular sample design captures spatial variation in altitude, we used a series of 5×5 km study areas overlaid on SRTM altitude values (version 2 “void filled”, downloaded from http://earthexplorer.usgs.gov/) distributed across the legal Brazilian Amazon (Figure S1). The legal Brazilian Amazon is a 5.06 million km^2^ area defined by the boundaries of nine Brazilian States: Acre, Amapá, Amazonas, Pará, Rondônia, Roraima, Tocantins, Mato Grosso, and Maranhão. The 5×5 km study areas included seven areas (hereafter “active research areas”, Figure 1, Table S1, Figure S3) that are part of the Brazilian Program for Biodiversity Research (“Programa de Pesquisa em Biodiversidade” – hereafter PPBio) [3], [22] and 1286 randomly selected areas (Figure S1, Figure S2). The randomly selected areas provided a representative sample of altitude across the entire legal Brazilian Amazon (Figure S1) and enabled us to establish that conclusions from the seven active research areas were more generally applicable.

The active research areas were included to provide a real-world sample of sites established to address research and biodiversity monitoring questions. PPBio has adopted a standardized survey system (“RAPELD” [3], [22], [27]) that uses regularly spaced plots along 5 km trails as its basic sample unit. One of the sample arrangements used within PPBio [3] is a 25 km^2^ area delimited by a series of 5 km trails that form a survey “grid” with sample plots regularly spaced at 1 km intervals (Figure S3). At the time of writing PPBio and partners has established ten 25 km^2^ research areas across Brazilian Amazonia. We included 7 of the 10 active research areas in our analysis based on two criteria: (i) with an established regular arrangement of sample plots (*n* = 30–31) distributed along a trail system, 1 km distant from each other (Figure S3), and (ii) with active research and data freely available (http://ppbio.inpa.gov.br/sitios).

In addition to the regular sample plots, the RAPELD grids also have “riparian plots” [3]. These riparian plots are installed wherever a trail crosses a waterbody and are used to survey the distinctive species found in riparian zones [30], [31], [32]. The distance between riparian plots within grids is not standardized, and only one grid (“Ducke”) has established riparian plots. Therefore, to enable comparison between grids we derived the probable locations of riparian plots within the other grids using standardized GIS processes (Figure S4).

### Data analysis

All statistical analyses were undertaken with the R language and environment for statistical computing [33], using base functions and functions available in the following packages: “ggplot2” [34], “gstat” [35], “mgcv” [36], “raster” [37], and “sp” [38].

We evaluated the adequacy of samples and their associated spatially interpolated values across the random and active research areas. Active research areas included three sample sizes (Table S1): (i) the existing arrangement of regular 1 km interval plots (*n* = 30–31), (ii) the same regular 1 km interval plots plus riparian plots (*n* = 45–52), and (iii) to examine the expected improvements generated by increasing sample size we also included locations if plots were to be established at regularly spaced 500 m intervals along the existing trails (*n* = 96),which also included the locations of the existing 1 km interval regular plots. Within the randomly selected areas, we simulated regularly arranged samples across a wider range of sample sizes (*n* = 4, 8, 16, 30, 60, 120). As the locations of the regular samples generated within each random area can differ (depending on the starting point), metrics for the samples and associated interpolations were generated for 10 iterations of each sample size within each random area. The mean value of the metrics for samples and spatial interpolations from the 10 iterations was used in all subsequent analysis.

Inverse Distance Weighted (IDW) spatial interpolations were calculated from the SRTM altitude values at the different sample sizes within each of the random and active research areas. Interpolations were generated for each area using a 90 m grid size that corresponded approximately to that of the original SRTM altitude values. The IDW approach was adopted as this spatial interpolation technique can be used with small sample sizes [9], [22], [26] and in our case provided similar results to Ordinary Kriging and Generalized Additive Model (GAM) interpolations (Figure S5, Figure S6).

To answer questions 1 and 2, the adequacy of samples and associated spatial interpolations was evaluated using the following methods. For question 1, we used the Kolmogorov-Smirnov (KS-test) as a measure of sample representativeness [26]. This was done by comparing the distribution of both sample and spatially interpolated altitude values with the original SRTM altitude values in both randomly selected and active research areas using the KS-test. For question 2 we examined the interpolated values from samples in random areas, using correlation as a measure of similarity between sample and population (i.e. original SRTM values) values, and the root mean square error (RMSE) to represent the accuracy of parameter estimation.

For question 3, the thresholds in all metrics were evaluated based on whether the parameter of interest reached stability and/or the chosen level of precision [39]. This was achieved using complementary approaches. Firstly, Generalized Additive Models (GAMs) [36] were used to model trends in the response of the different metrics to sample size and standard deviation (SD). Secondly, to identify thresholds (i.e. break-points) where sample size and SD influenced the responses we used the conditional inference tree method [40], [41], implemented in the “partykit” package [42]. Although various regression-tree methods are available (e.g. CART, random forest, etc.) we assume that results from different methods will be similar for simple cases such as ours.

## Results

### Sample and Interpolation Representativeness

Mean SRTM altitude within 5×5 km areas of the 5.06 million km^2^ legal Brazilian Amazon was 159.5 m (range 0–1216 m). Mean altitude in the randomly selected areas (159.3) was similar to this overall mean, but the greatest mean value in the randomly selected areas was lower at 683 m. Altitude heterogeneity (SD) ranged from 0 to 466 across 5×5 km areas of the 5.06 million km^2^ legal Brazilian Amazon (Figure S1). However, 90% of areas had SD values < = 40, which corresponds to a range in altitude of ≈210 meters [mean of range values from the randomly selected areas with SD of 40 (*n* = 7)]. Our sample of seven active research areas (SD ranging from 4.8 to 40.2, Table S1) therefore represents the meso-scale altitudinal heterogeneity found across an area of approximately 5 million km^2^.

**Figure 2:**
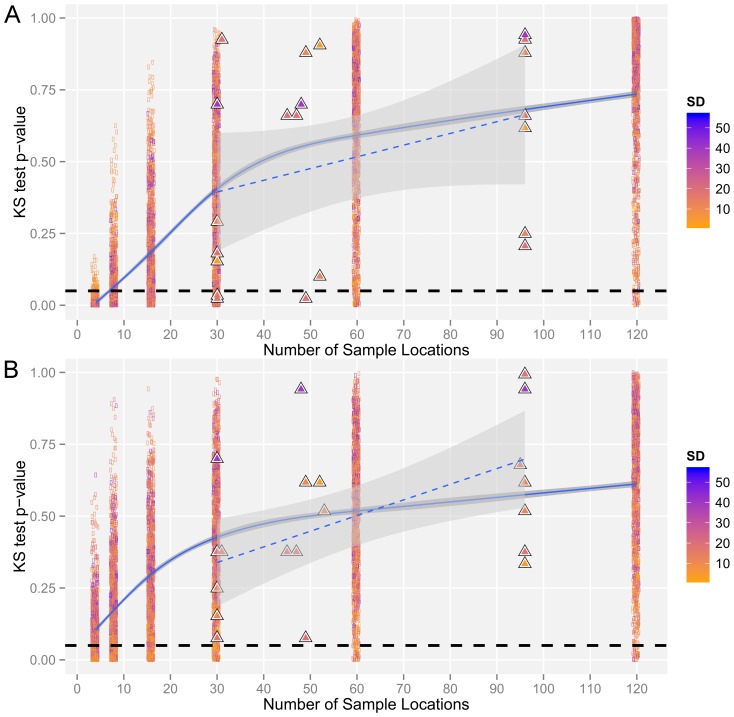
Comparison of sample representation across 25 km^2^ of Amazonian forest. The sample representativeness of altitude values was determined in two sets of 5×5 km sample areas [1286 randomly selected (rectangles) and seven active research areas (triangles)] across the Brazilian Amazon. Within each area, the representativeness of (a) samples and (b) their corresponding IDW interpolations were evaluated. The distribution of values was compared with that of the area altitude (SRTM DEM) values using the Kolmogorov-Smirnov (KS) test. Increasing *p*-values denote more similar distributions (i.e. samples/interpolations are more representative). The blue lines (solid  =  randomly selected and dashed  =  active research areas) and shaded areas are the mean value and 95% confidence intervals from a GAM model illustrating the trend in representativeness with increasing sample size. The filled colors show the standard deviation (SD) of altitude values within each of the different sample areas. The black dashed horizontal line shows a *p*-value of 0.05.

The Kolmogorov-Smirnov (KS) tests showed that a regular arrangement of samples could represent the distribution of altitude values within our study areas (Figure 2, Figure S7, Figure S8). For the active research areas, the KS-test showed that the distribution of altitude values in the samples from two areas [FLONA do Amapá (*n* = 30), and Virua (*n* = 30 and *n* = 49)] were (marginally) significantly different from the distribution of altitude values within grids (Figure 2a, Figure S8). Although statistically different, visual inspection of back-to-back histograms showed that even these smaller samples (*n* = 30, *n* = 49), appeared to capture the distribution of altitude values in these two areas with long-tailed distributions i.e. differences appear to be caused by variation in the distribution of the more extreme values (Figure S8).

### Spatial interpolation: similarity and accuracy

**Figure 3:**
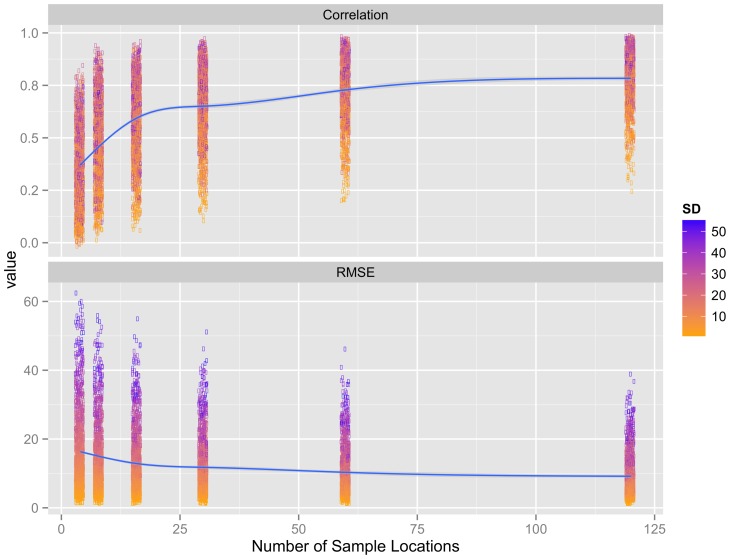
Trends in interpolation adequacy with increasing sample size. IDW (Inverse Distance Weighted) models were used to interpolate altitude (SRTM DEM) based on values from regularly distributed sample locations (*n*: 4, 8, 16, 30, 60 and 120) within 1286 areas (5×5 km) across the Brazilian Amazon. Interpolation adequacy was evaluated using root mean square error (RMSE) and correlations calculated from the interpolated values in relation to the original SRTM altitude values. Lines and shaded areas are mean values and 95% confidence intervals from GAM models illustrating trends with increasing sample size. The filled colors show the standard deviation (SD) of altitude values within each of the different sample areas.

Both sample size and standard deviation (SD) strongly influenced the similarity and accuracy of spatial interpolations (Figure 3). The similarity between interpolations and the original altitude values increased rapidly with sample size (Pearson correlation mean values increased from 0.35 to 0.57, at *n* = 4 to *n* = 16 samples respectively) after which correlations continued to increase but at a lesser rate, reaching a mean value of 0.78 at a sample size of 120.

### Thresholds

Conditional inference tree analysis revealed differences in the influence of sample size and sample standard deviation on thresholds (break-points) in sample accuracy (representativeness, similarity and accuracy - Figures S9–S13). This analysis showed that sample size was the most important determinant of sample representativeness (KS-test *p*-values, Figure S9), with the first, second and third splits all due to sample size. The first split of this tree was at *n* = 16, grouping the smaller sample sizes (*n* = 4, 8, 16), which, independent of SD, were less representative (lower *p*-values) compared with the larger sample sizes (*n* = 30, 60, 120). The GAM model (Figure 2a, *p*<0.00001, deviance explained  = 65.2%) showed that the sample KS-test *p*-values did not stabilize within the range of sample sizes examined, but did stabilize at SD values ≥9.

Generally, the representativeness of interpolated values showed a similar pattern to sample values (Figure 2). This similarity was also reflected in the first two splits of the interpolated KS-test *p*-value classification tree (Figure S10), which were also based on sample size, with the first split again at *n* = 16. Overall, 5 of the 9 sample KS-test tree inner nodes were based on sample size, and 5 of the 11 interpolation KS-test tree inner nodes were based on sample size (*p*<0.001 for all sample and interpolation inner nodes). However, GAM models reinforced differences between sample and interpolated values, with interpolated values statistically representative (on average) even at the smallest sample size (*n* = 4, Figure 2b, *p*<0.00001, deviance explained  = 49.3%).

With respect to the similarity and accuracy of spatially interpolated values, the conditional inference trees (Figures S11, S12) agreed with GAM results (Figure 3), showing that sample size was a more important determinant of correlations than for RMSE values. The GAM model (Figure 3, *p*<0.00001, deviance explained  = 58.5%), showed that correlations started to stabilize after *n* = 16 (increasing little after *n* = 60) and stabilized at SD values >15. As was found for the KS-test *p*-values (both sample and interpolation), the first split of the correlation classification tree was at *n* = 16 (Figure S11), whereas the first split of the RMSE tree was at SD = 19.77. The GAM model (Figure 3, *p*<0.00001, deviance explained  = 86.1%) showed that RMSE values stabilized after *n* = 16, but continued to increase across the range of SD values examined. Although sample size was not as important for RMSE values, a sample size of 16 was also identified at the 3^rd^ and 4^th^ splits of the RMSE tree (Figure S12). Overall, 5 of the 10 correlation tree inner nodes were based on sample size, whereas only 3 of the 11 RMSE tree inner nodes were based on sample size (*p*<0.001 for all correlation and RMSE inner nodes).

The difference in the importance of sample sizes and SD for correlations and RMSE was also confirmed by visual inspection (Figure 3), which showed that RMSE values tended to increase with SD at any given sample size, whereas this relationship was not as clear for correlations. Although there was a significant association between SD and interpolation similarity and accuracy, visual inspection of the IDW estimate maps showed that the meso-scale trends in altitude were represented well across the research grids (with SD values between 4 and 40) using a regularly arranged sample size of 30 (Figure S13).

## Discussion

Our findings indicate that a regular arrangement of 30 sparse samples represent meso-scale altitude in 25 km^2^ Amazonian areas. We also found that the associated interpolations are statistically similar to values at a much more detailed resolution (≈90 m). We discuss these findings and their potentially wide reaching applications in relation to obtaining samples necessary for monitoring biodiversity changes.

### Sample and Spatial Interpolation Representativeness

Accurate and unbiased samples are needed to enable derivation of any of the myriad metrics used by researchers to examine ecological patterns and processes and to monitor and predict changes in biodiversity [1]. Our understanding of biological diversity within tropical biomes usually comes from sparse samples [1], [3], [4]. The continued debate regarding species richness [43], perhaps the most commonly used indicator in studies investigating biodiversity and conservation (not to mention ecological and macroecological research) highlights how sample adequacy affects conclusions even for the most simple biodiversity metrics.

Finding that regularly arranged samples are representative of meso-scale altitude values was expected as this arrangement is known to be efficient [10]. What was surprising was that even relatively small sample sizes (*n* = 8) were statistically representative (i.e. mean KS-test *p* value >0.05). However the considerable variation around the mean values suggests that such low sample sizes may not be generally reliable. This variation in sample representativeness was only partly explained by differences in sample size and standard deviation (65.2% of the GAM model deviance explained). As shown by numerous previous studies [9], [10], it therefore seems likely that additional factors such as pre-existing gradients and directionality (i.e. anisotropy) will also influence sample representativeness [9].

A more detailed examination of the research grids showed that statistical representativeness appeared to be strongly influenced by the frequency distribution of the sample values. Samples from research grids with longer tailed distributions (i.e. with “extreme”/“unusual” values) were less representative. This finding was expected as it is widely recognized that studies focusing on rare/extreme events/values require the development of specific sample arrangements and/or analytic techniques [44], [45]. Nevertheless, visual examination of mapped values and the histograms comparing sample and original distributions suggested that the regular samples captured meso-scale patterns in at least some of these statistically “unrepresentative” cases.

The mapped research grid interpolations also illustrated how the statistical representativeness of the samples translated into predicting the real-world local-scale (90 m) spatial patterns in altitude. Indeed our findings suggest that a regular arrangement of ≥4 samples can (on average) be considered as statistically representative within the randomly selected areas. However, there was considerable variation around the mean. This variation shows that such small sample sizes do not necessarily translate into samples that are appropriate for the detection of either local or meso-scale patterns.

### Spatial interpolation: similarity and accuracy

We show that regular meso-scale samples generate interpolations representative of a response measured at the local scale (90 m). Even at small sample sizes (<30) the interpolations were accurate (mean RMSE <20 m) and representative (mean KS-test *p* value >0.05). However, correlations with the original SRTM values were weak (*r*<0.5) at these smaller sample sizes. Additionally the accuracy of interpolated samples was strongly dependent on the sample standard deviation at all sample sizes. Such strong dependence on sample standard deviation means that even with the most intensive sampling (*n* = 120) there was a degree of uncertainty in the accuracy of such local scale predictions in the more heterogeneous areas (SD values greater than 10). While it is clearly important to acknowledge such uncertainty, visual examination of the maps generated showed that interpolations from sample sizes of 30 captured the meso-scale distribution in altitude even in the more heterogeneous areas (SD>10).

The representativeness, similarity and accuracy of interpolated values means that it is theoretically possible to compare metrics derived from these interpolations with those obtained from any other sample size/arrangement at similar scales that adequately represent the variable of interest. The ability to generate accurate interpolations also means that we are able to adopt the (typically more powerful) statistics used for continuously sampled areas [9]. The use of interpolated values can therefore provide an option to integrate/compare/contrast results from studies that adopt different sample designs and sizes. As demonstrated by recent reviews [46], [47] and case studies [48], we would expect the integration of results from different studies to generate substantial improvements in relation to the insights and potential applications of the data generated. Such improvements are also likely to generate financial economies [46], [48]. The economies arising from the ability to integrate data from different sources are particularly relevant in the case of payments for ecosystem services where indicators and monitoring protocols are likely to vary spatially with context and location and temporally with the emergence of future threats [46], [49], [50], [51].

### Thresholds

Classification-tree analysis showed that sample sizes equal to or greater than 30 were an important threshold for representativeness, accuracy and similarity. In combination with the GAM results, these findings show that a regularly arranged sample size of 30 presents values that are representative of, accurate, and similar to the original “population” (i.e. meso-scale altitude). The thresholds identified broadly agree with established rules of thumb for a sample size of 30 in spatial ecology [9] and a sample size of 50 as the computational minimum for geostatistical analysis [26].

But how representative are these sample size thresholds for biodiversity monitoring? Our findings are based on the spatial variation in altitude and the underlying assumption is that there will be similar variation in meso-scale “biodiversity” patterns. Defining biodiversity was one of the questions addressed in a classic issue of the Philosophical Transactions of the Royal Society B|Biological Sciences (July 29, 1994, vol. 345 (1311): http://rstb.royalsocietypublishing.org/content/345/1311.toc). After 20 years, the increasing number and complexity of biodiversity metrics (e.g. [7], [48]) suggests that we are not much closer to a unifying definition for quantifying biodiversity. Lacking a clear understanding of how to quantify biodiversity means that extrapolating conclusions from meso-scale altitude to meso-scale tropical forest “biodiversity” is challenging. However, to examine whether the thresholds identified are likely to be generally applicable for biodiversity monitoring we can compare the meso-scale variation in altitude with variation in some of the species and community responses documented from tropical forests.

Studies within one of the PPBIO research grids (“Ducke”) provide a detailed multi-taxa assessment of biodiversity [52]. Although there are exceptions, previous studies (e.g. [53], [54], [55], [56]), and the more general overview provided in [3]), show that meso-scale patterns in the vast majority of species and communities vary at similar scales to altitude within this area (altitude mean ± SD (range): 91±18.9 (53–127)). In other words, 2 to 3 fold variations in meso-scale richness and diversity are relatively common within Ducke but 100 fold variations are rare. The fact that variation in the community diversity of several groups is not explained by topographic variation i.e. slope (see Table 1 of [52]), is not necessarily critical in this case. The more important point is that at meso-scales the spatial variation of species and communities is often similar to or less than the variation in altitude [53], [54], [55], [56]. Therefore, we can expect that the same sample size thresholds we identified for representing altitude should also be representative for quantifying numerous meso-scale biodiversity responses.

There are obvious limitations to the thresholds identified. We only considered the sample representativeness in areas with a standard deviation ≤56, corresponding to 95% of the legal Brazilian Amazon. The thresholds identified are unlikely to be applicable in areas where altitude varies more, such as the Andean highlands. Although it is theoretically possible to establish regular 25 km^2^ sample grids in highly heterogeneous areas, this is unlikely to be a cost effective approach in locations with strong preexisting gradients/strata (e.g. highly heterogeneous topographic variation/land cover types). In such situations we would expect that some sort of stratified sample scheme should be the most cost effective [4], [57].

### Practical implications

But what is the optimum sample design for monitoring Amazon biodiversity? The answer to this question is location and context specific and therefore lies with individual researchers/project managers. Within the context of biodiversity monitoring, study designs must provide the most information for the lowest price [1], [4] i.e. meet statistical requirements and be practically and logistically possible [1], [4]. Some consider a regular arrangement to be unnecessarily “demanding” in terms of sampling effort. For example, Gardner [4] highlights that “the major disadvantage of a uniform-grid approach is that it demands a high level of sampling effort”, but the same author describes a stratified-random approach using considerably more effort than 30 sample points [57]. It is clear that a regular design would probably cost more to implement than the stratified approach to answer the questions in [57]. But the efforts described in [57] suggest that a sample size of 30 across a 25 km^2^ area is well within the logistic and financial “norm” of Amazon research projects. An often overlooked benefit of standardized samples that adequately represent the target response is that it is possible to directly compare and contrast values of interest. For example, it is possible to directly compare intuitive and easily communicable metrics, such as number of individuals (*N*) or number of species (*S*) to represent biodiversity. The ability to communicate and inform results is one of the advantages of standardized samples that is, however, rarely included in comparisons of sampling designs [1].

In cases where strata can be identified (the logical pre-requisite for stratification), there will probably be sufficient information (e.g. describing magnitude of expected response, magnitude of possible confounding effects) to develop robust sampling designs to meet research objectives. Land use change coupled with the presence of multi-jurisdictional landscapes, (where it is often impossible to assume a regular design as some land-owners will not permit access (T. Gardner pers. com.)) means that there is a large area within the legal Brazilian Amazon where a 25 km^2^ regular sampling grid is unlikely to be practical. Yet at the same time, Amazon deforestation is decreasing and there remain vast tracts of forest cover across the Brazilian Amazon [58]. For example the Amazon Region Protected Areas (ARPA) program is a network that includes over 90 protected areas, and covers 51 million hectares [59]. Therefore, there remains a substantial area where knowledge to inform sample stratification for biodiversity monitoring is unavailable (e.g. unknown future threats, poorly described response/predictor variables, multiple response variables) and stratification is therefore unlikely to be a viable option [3], [27], [28].

We are not advocating one arrangement over another, and are merely demonstrating the adequacy of regularly arranged sample sizes. Indeed, a regular arrangement is unlikely to be suitable for a variety of relevant ecological questions, such as studies examining rare species or the density of large canopy trees [60]. However, it is a relatively simple task to develop more detailed studies using a subsample of the logistics provided by a broader scale sample arrangement. For example, many researchers use only a fraction of a 25 km^2^ PPBio grid to conduct a wide variety of relevant studies [3]. It is also worth noting that a recent study showed that common indicators are more likely to be appropriate compared with rare species indicators within the context of monitoring biodiversity for environmental service payments [5].

Sommerville et al. (2011) [5] present a framework for effectively monitoring environmental services where it is necessary to make three major decisions: (i) which indicators to monitor (e.g. a species of concern/threats/positive actions), (ii) how to monitor the chosen indicators (e.g. ground based, remote sensing or a combination) and (iii) how to use the monitoring results to determine payments (e.g. presence-absence of an indicator/the trend of the indicator over time/detecting differences between monitored sites/achievement of targets). These choices will dictate the most adequate sampling design for monitoring biodiversity within any given context/locality. Decades of research present detailed evaluations of how to derive spatially optimal sampling strategies [10], [61], [62], [63] and rather than seek to optimize for the general “biodiversity monitoring” case (which never exists) we show that metrics obtained from a regular sample are likely to be both widely applicable and comparable across ≈5 million km^2^ of the legal Brazilian Amazon.

## Conclusions

Although not all biodiversity indicators are directly related to altitude in the Amazon, in the majority of situations, it will at least be necessary to model and/or control for the effects of altitude in both within and between site comparisons. The ability to represent meso-scale altitudinal variation with a regular arrangement of 30 samples provides strong support for the idea that such an arrangement and sample size will be adequate for a wide range of present and future biodiversity monitoring challenges. Knowing that samples/interpolations provide a reasonable representation of meso-scale altitude values means that we are able to reliably use such samples to examine whether within and between-site differences in patterns of biodiversity indicators are or are not driven by altitudinal/topographical variation. The capability to compare interpolated values with those derived from other representative sampling designs is also likely to facilitate both comparison across studies and communication between researchers and managers.

## Supporting Information

Figure S1 Random sample areas.
(DOC)

Figure S2 Correlation and RMSE of interpolated altitude values.
(DOC)

Figure S3 Distribution of sample plots within seven active research areas.
(DOC)

Figure S4 Obtaining locations of river-trail intersections.
(DOC)

Figure S5 Mapped comparison of IDW, Kriging and GAM interpolations.
(DOC)

Figure S6 Comparison of IDW, Kriging and GAM interpolations.
(DOC)

Figure S7 Sample “representativeness.”
(DOC)

Figure S8 Sample “unrepresentativeness.”
(DOC)

Figure S9 Sample KS-test conditional inference tree.
(DOC)

Figure S10 IDW KS-test conditional inference tree.
(DOC)

Figure S11 IDW correlation conditional inference tree.
(DOC)

Figure S12 IDW error conditional inference tree.
(DOC)

Figure S13 Mapped IDW estimates.
(DOC)

Table S1 Active research areas.
(DOC)
