# Supplementary material for: Towards Monitoring Biodiversity in Amazonian Forests: How Regular Samples Capture Meso-Scale Altitudinal Variation in 25 km2 Plots
Source: PLoS One. 2014 Aug 29;9(8):e106150. doi: 10.1371/journal.pone.0106150 (PMC4149511; doi:10.1371/journal.pone.0106150)
Supplement: Figure S1 — Random sample areas. (DOC) [file pone.0106150.s001.doc]

S1 Random sample areas

We used a random selection to obtain a representative sample of altitude across the legal Brazilian Amazon. We divided a polygon of the legal Brazilian Amazon (≈5.06 million km^2^, shapefile downloaded from: <http://www.dpi.inpe.br/amb_data/Shapefiles/UF_AmLeg_LLwgs84.zip> ) into 5 x 5 km cells (“virtual” study areas), which resulted in a total of 140,680 cells. To select study areas these polygons were then overlaid on the SRTM DEM raster. To avoid analytic edge effects (for example where the two data sources did not overlap) we excluded those cells within 30 km of the polygon limits. We also excluded all 5 x 5 km cells with SRTM standard deviation values < 1 (i.e. “flat” areas such as rivers). We then randomly selected 1356 (1%) of the remaining cells (Fig S1). We consider altitude values of this sample to be representative as they both followed the same distribution as (Kolmogorov-Smirnov test, D= 0.4, *p*=0.06, Figure S1) and were highly correlated with (Spearman correlation, r = 0.8, *p*<0.0001) those found across the entire legal Brazilian Amazon.

The sample of 1356 randomly selected areas was used to evaluate the influence of sample size and heterogeneity (standard deviation - SD) on meso-scale interpolations. Visual examination showed that the extreme SD values seemed to be obscuring trends (in mean values) particularly in the patterns of RMSE (Figure S2). We therefore retained 95% of sample areas (n=1286), excluding areas with mean SD values > 56 (corresponding to the 95% quantile of the sample SD values).

| 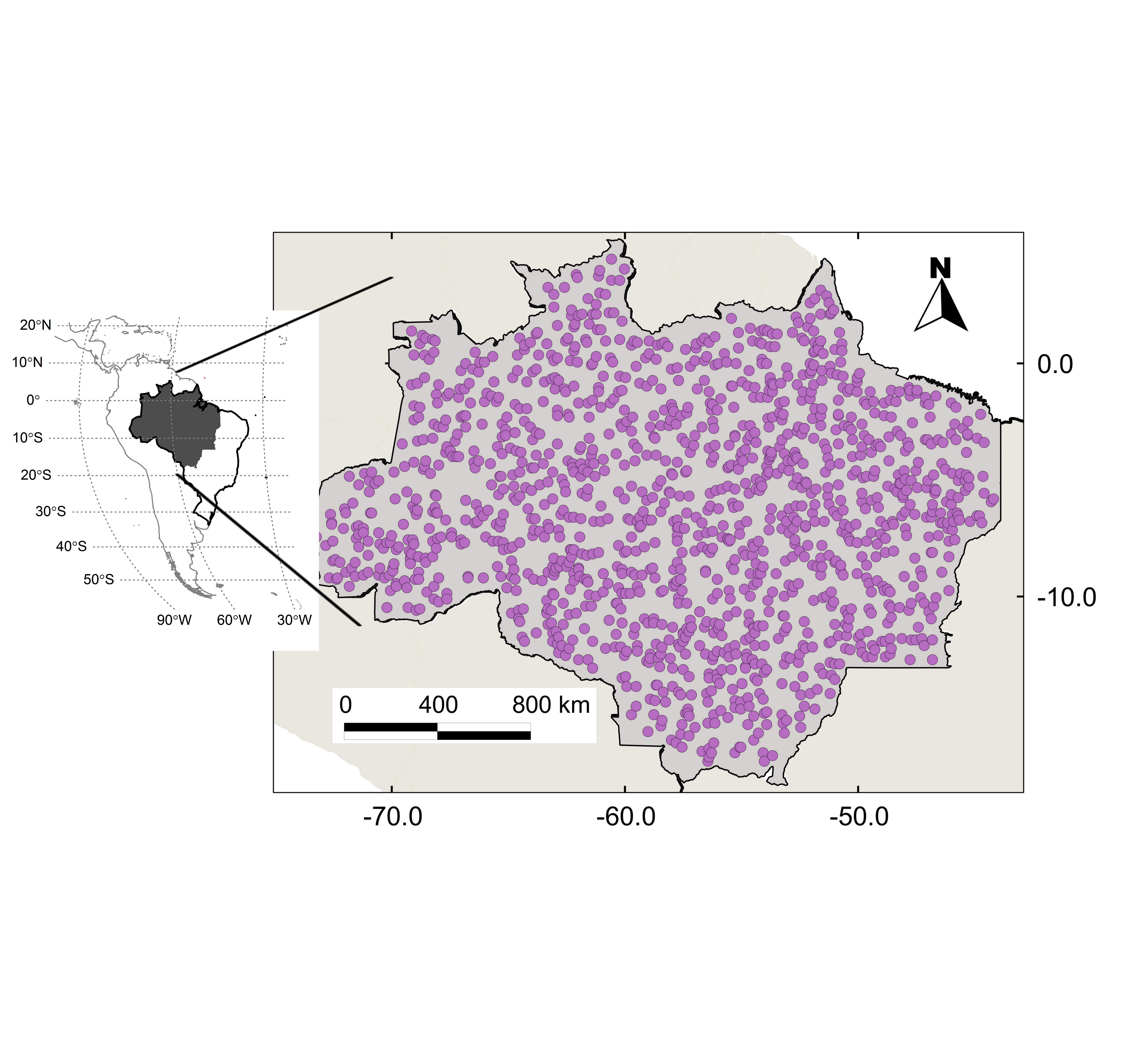 |  |
| --- | --- |

Figure S1 Map of the legal Brazilian Amazon showing the location of 1356 sample areas (5 x 5 km) used to evaluate the influence of sample size on meso-scale interpolations. The back-to-back histogram shows the distribution of altitude values across the randomly selected sample areas (n=1356) and the map area (legal Brazilian Amazon).
