# Supplementary material for: Towards Monitoring Biodiversity in Amazonian Forests: How Regular Samples Capture Meso-Scale Altitudinal Variation in 25 km2 Plots
Source: PLoS One. 2014 Aug 29;9(8):e106150. doi: 10.1371/journal.pone.0106150 (PMC4149511; doi:10.1371/journal.pone.0106150)
Supplement: Figure S2 — Correlation and RMSE of interpolated altitude values. (DOC) [file pone.0106150.s002.doc]

S2 Correlation and RMSE of interpolated altitude values


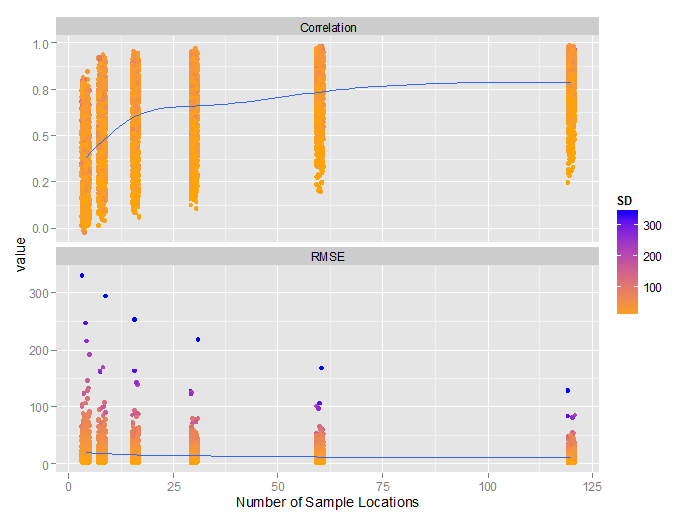


Figure S2 Trends in interpolation adequacy with increasing sample size in 1356 randomly selected areas. IDW (Inverse Distance Weighted) models were used to interpolate altitude (SRTM DEM) based on values from regularly distributed sample locations (n: 4, 8, 16, 30, 60 and 120) within 1356 areas (5 x 5 km) across the legal Brazilian Amazon. Interpolation adequacy was evaluated using root mean square error (RMSE) and correlations calculated from the interpolated values in relation to the original SRTM altitude values. Lines and shaded areas are mean values and 95% confidence intervals from GAM models illustrating trends with increasing sample size. The filled colors of the circles show the standard deviation (SD) of altitude values within each of the different sample grid areas.
