# Supplementary material for: Towards Monitoring Biodiversity in Amazonian Forests: How Regular Samples Capture Meso-Scale Altitudinal Variation in 25 km2 Plots
Source: PLoS One. 2014 Aug 29;9(8):e106150. doi: 10.1371/journal.pone.0106150 (PMC4149511; doi:10.1371/journal.pone.0106150)
Supplement: Figure S3 — Distribution of sample plots within seven active research areas. (DOC) [file pone.0106150.s003.doc]

| 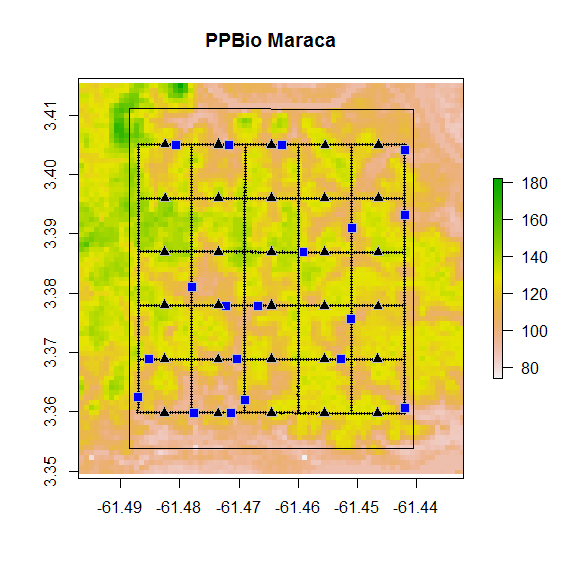 | 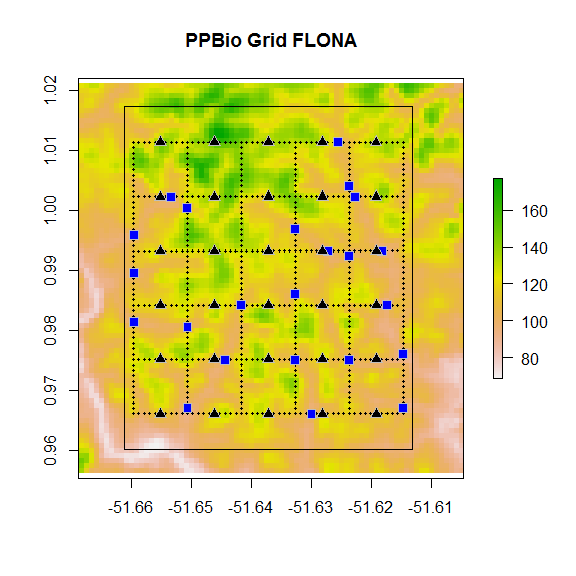 | 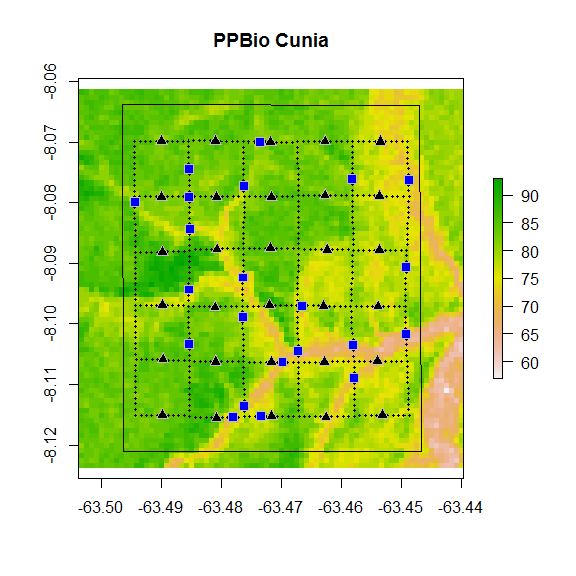 |
| --- | --- | --- |
| 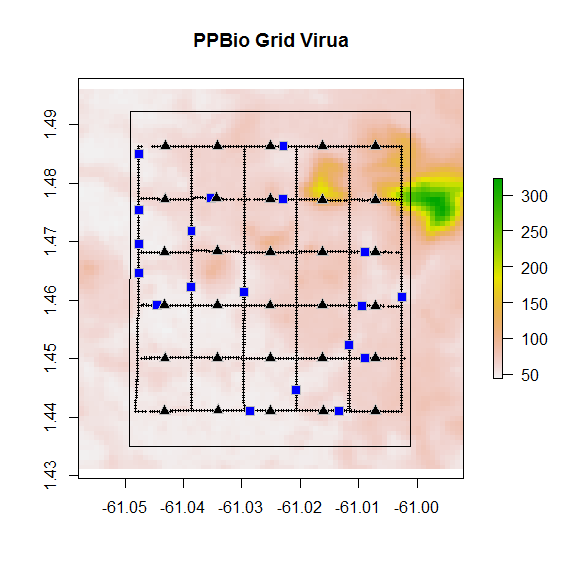 | 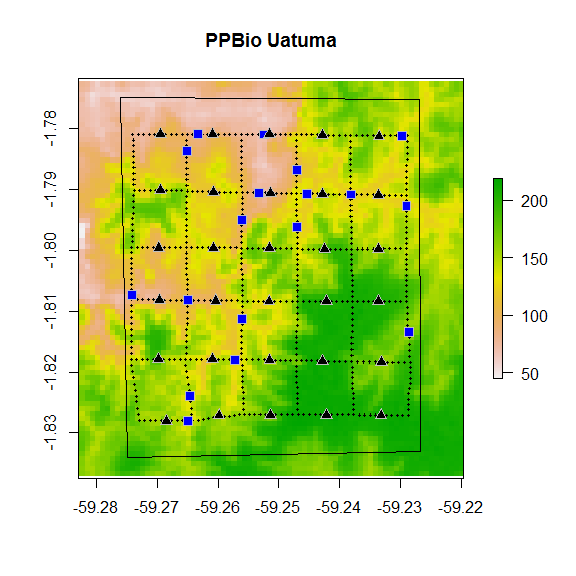 | 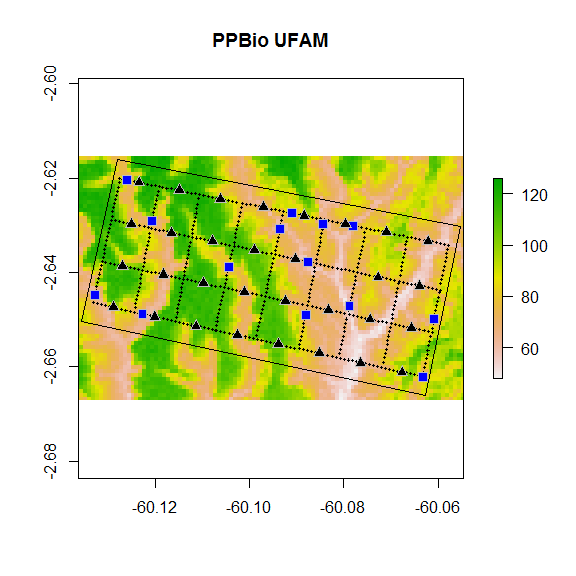 |
| 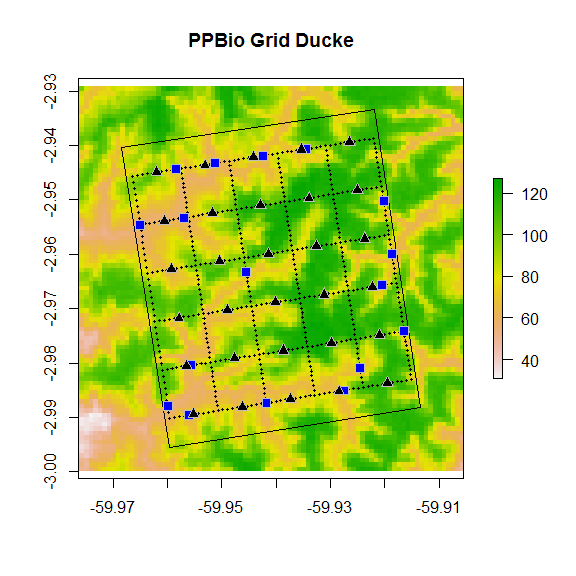 |  |  |

Figure S3 Distribution of sample plots within seven active research areas. Maps show the location of regular plots (solid black triangles, n= 30 – 31) established along the trails (dotted lines) at 1km intervals and plots located where streams intersect trails (solid blue squares, n= 14 – 23).
