# Supplementary material for: Towards Monitoring Biodiversity in Amazonian Forests: How Regular Samples Capture Meso-Scale Altitudinal Variation in 25 km2 Plots
Source: PLoS One. 2014 Aug 29;9(8):e106150. doi: 10.1371/journal.pone.0106150 (PMC4149511; doi:10.1371/journal.pone.0106150)
Supplement: Figure S4 — Obtaining locations of river-trail intersections. (DOC) [file pone.0106150.s004.doc]

S4 Obtaining locations of river-trail intersections

To obtain “river” sample locations in the active research areas without established “river” sample points (Cuniã, FLONA Amapa, Maraca, UFAM, Uatumã and Virua) we derived probable locations of the river-trail intersections within a GIS. This was done by using the SRTM DEM to generate river channel networks using standard GIS processes. Deriving river channel networks is a feature common to the majority of GIS software [1]. We used SAGA (System for Automated Geoscientific Analyses) GIS (<http://www.saga-gis.org/en/index.html>), for data preprocessing and channel network derivation (modules: “Fill Sinks (Wang & Liu)” and “Channel Network and Drainage Basins”). SAGA is freely available from: http://sourceforge.net/projects/saga-gis/files/.

The channel networks were then overlaid on the trail system to obtain locations where the river channels intersected the grid trails. The GIS process was evaluated against Ducke river locations (trail points where rivers were known to intersect the grid system). For this analysis we used the intensively surveyed 8 x 8 km grid at Ducke. The GIS process generated 21 (76.3%) of the 38 river locations within the channel network (Figure S4). As such we consider the sample sizes obtained using this GIS process representative of the river/trail intersections within a site.

| 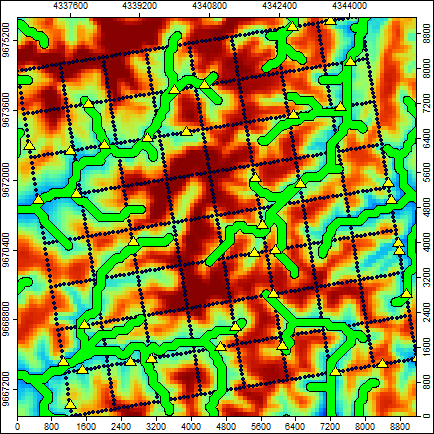 |
| --- |

Figure S4. Evaluation of river/trail intersections obtained using GIS. Showing the river channels derived using standard GIS processes (solid green lines), locations of the established trail system (blue points) and the locations of the established river plots (yellow triangles).

References

1. DeMers, Michael N. 2009. GIS for Dummies. John Wiley & Sons.
