# Supplementary material for: Towards Monitoring Biodiversity in Amazonian Forests: How Regular Samples Capture Meso-Scale Altitudinal Variation in 25 km2 Plots
Source: PLoS One. 2014 Aug 29;9(8):e106150. doi: 10.1371/journal.pone.0106150 (PMC4149511; doi:10.1371/journal.pone.0106150)
Supplement: Figure S5 — Mapped comparison of IDW, Kriging and GAM interpolations. (DOC) [file pone.0106150.s005.doc]

S5 Mapped comparison of IDW, Kriging and GAM interpolations

Our analysis was based on inverse-distance-weighted interpolations. As there are a number of interpolation techniques available we examined whether the patterns revealed by IDW interpolations were consistent with those from other techniques. IDW interpolations were compared with those from generalized additive models (GAMs – non-parametric smoother) and ordinary Kriging. Visual inspection of mapped interpolations (Fig S5.1) showed that patterns were consistent across the three techniques (Pearson’s correlation >0.94 in all pairwise comparisons of interpolations from the three techniques) for any given sample size (n=30, 47, 96). Additional comparison with the original SRTM values in the seven active research areas showed that interpolations from all three techniques had similar levels of correlation and error (Fig S6 – note strongly overlapping 95% confidence intervals). Although all three techniques resulted in similar values it is interesting to note that for the sample sizes tested IDW interpolations showed the strongest correlations (mean Pearson values: 0.77, 0.75, 0.74 for IDW, GAM and OK respectively) and lowest error (mean RMSE values: 10.24, 10.68, 10.78 for IDW, GAM and OK respectively).

|  |
| --- |
|  |
|  |

Figure S5.1 Mapped altitude in 25km^2^ of Amazonian forest. Illustrative example from one area (Ducke), with rows presenting interpolations from 3 different sample sizes (n=30, 47, 96) and crosses show locations of sample points. Columns show the interpolation technique [original altitude (“ALT.SRTM”) and estimates from three interpolation models (IDW= Inverse Distance Weighted, GAM=generalized additive model, OK = Ordinary Kriging)].
