# Supplementary material for: Towards Monitoring Biodiversity in Amazonian Forests: How Regular Samples Capture Meso-Scale Altitudinal Variation in 25 km2 Plots
Source: PLoS One. 2014 Aug 29;9(8):e106150. doi: 10.1371/journal.pone.0106150 (PMC4149511; doi:10.1371/journal.pone.0106150)
Supplement: Figure S6 — Comparison of IDW, Kriging and GAM interpolations. (DOC) [file pone.0106150.s006.doc]

Figure S6. Comparison of IDW, Kriging and GAM interpolations


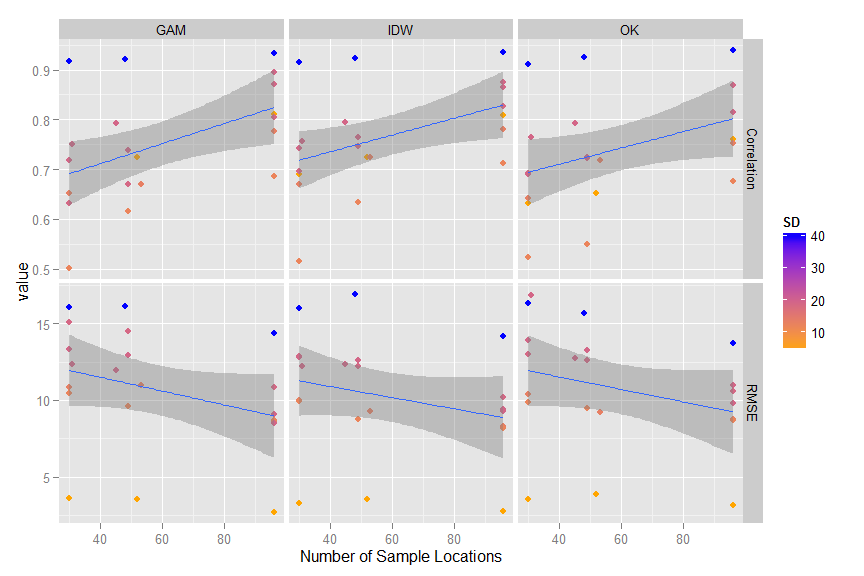


Figure S6 Comparison of interpolation techniques across seven active research areas. Three models (GAM=generalized additive model, IDW= Inverse Distance Weighted, OK = Ordinary Kriging) were used to interpolate altitude (SRTM DEM) based on values from sample locations (n= 30-31, 45-53, and 96) in seven areas (25km^2^). Sample adequacy was evaluated using two metrics (figure rows): correlation and root mean square error (RMSE). These evaluation metrics were calculated from the model estimates in relation to the original SRTM altitude values. Smooth lines and shaded areas are mean values and 95% confidence intervals from GAM models illustrating trends in sampling adequacy across each technique/metric combination.
