# Supplementary material for: Towards Monitoring Biodiversity in Amazonian Forests: How Regular Samples Capture Meso-Scale Altitudinal Variation in 25 km2 Plots
Source: PLoS One. 2014 Aug 29;9(8):e106150. doi: 10.1371/journal.pone.0106150 (PMC4149511; doi:10.1371/journal.pone.0106150)
Supplement: Figure S7 — Sample “representativeness.” (DOC) [file pone.0106150.s007.doc]

S7 Sample “representativeness”

The distribution of values obtained using logistically feasible sample arrangements (n= 30 - 96) was compared with the distribution of values within the sample area (the active research areas) using Two-sample Kolmogorov-Smirnov test. This test enables us to examine whether the sample distribution differed from the distribution of values within the areas (i.e. increasing p values indicate increasing similarity between the two distributions). The back to back histograms presented in Figure S7 show the sample representativeness across an illustrative subsample of areas with differing altitude heterogeneity: low (Cuniã, SD = 4.8), intermediate (Ducke PPBio, SD=18.9) and high (Uatumã, SD = 40.2).

| 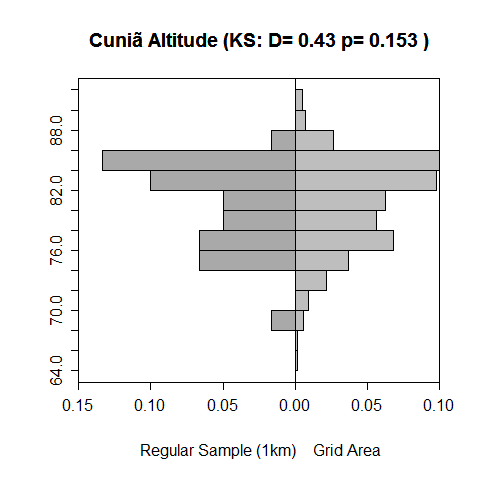 | 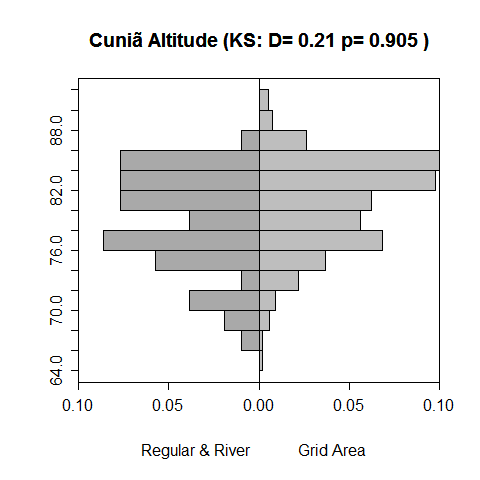 | 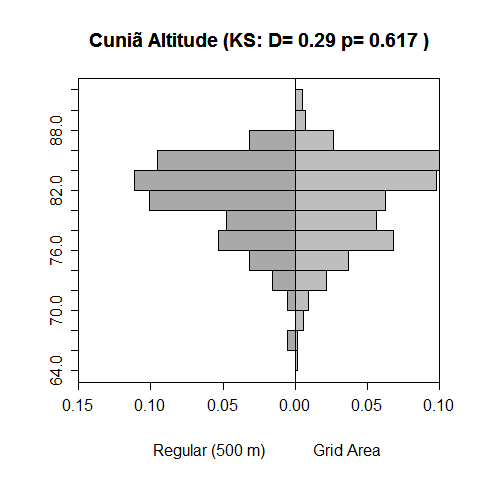 |
| --- | --- | --- |
| 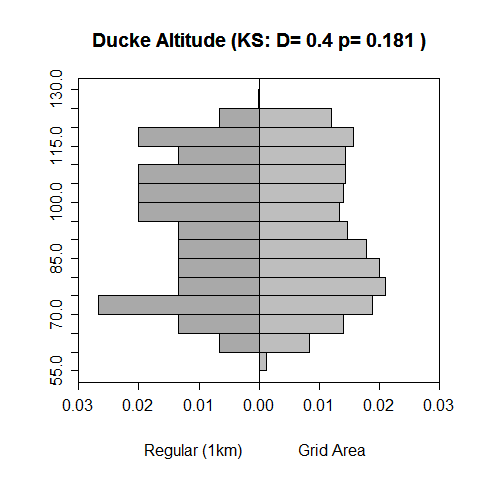 | 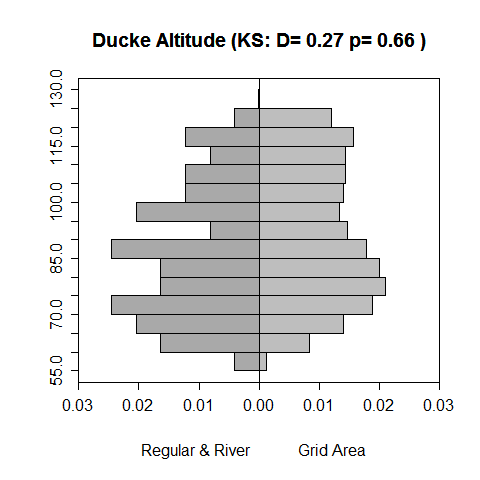 | 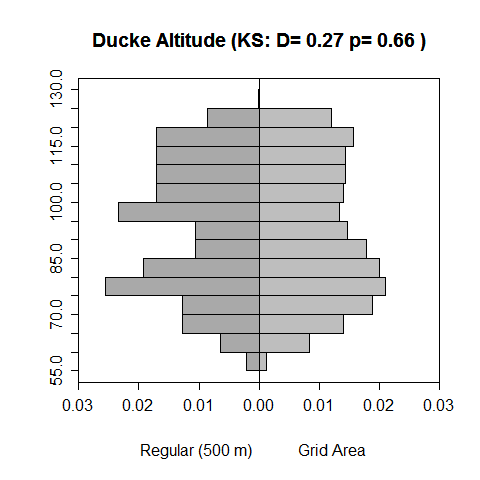 |
| 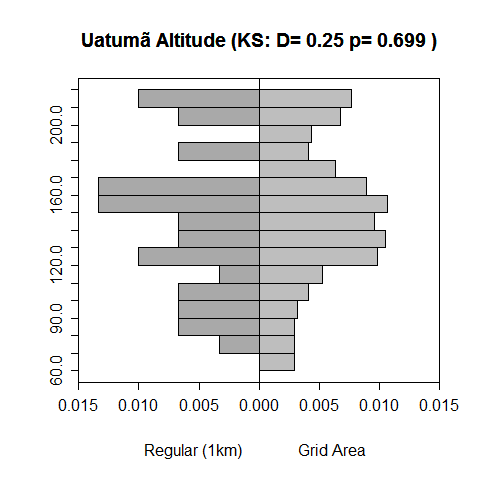 | 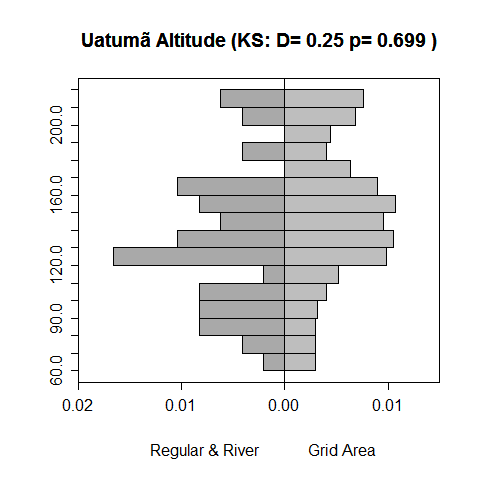 | 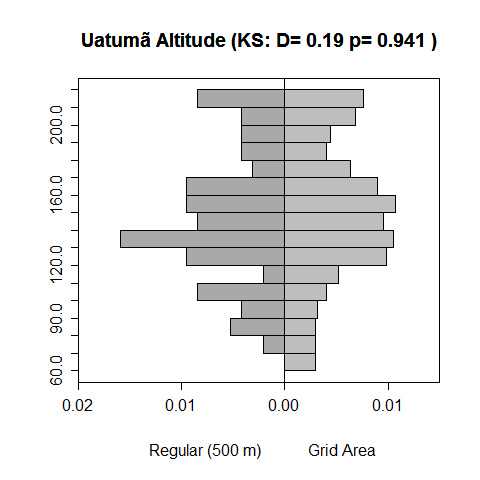 |

Figure S7. Sample representativeness across an illustrative subsample of grid sample areas. Grids with different variation (SD) in altitude low (Cuniã, SD = 4.8), intermediate (Ducke, SD=18.9) and high (Uatumã, SD = 40.2). Columns show comparison at different sample sizes (*n*=30, 46-49, 96).
