# Supplementary material for: Towards Monitoring Biodiversity in Amazonian Forests: How Regular Samples Capture Meso-Scale Altitudinal Variation in 25 km2 Plots
Source: PLoS One. 2014 Aug 29;9(8):e106150. doi: 10.1371/journal.pone.0106150 (PMC4149511; doi:10.1371/journal.pone.0106150)
Supplement: Figure S8 — Sample “unrepresentativeness.” (DOC) [file pone.0106150.s008.doc]

Figure S8. Sample “unrepresentativeness”.

| 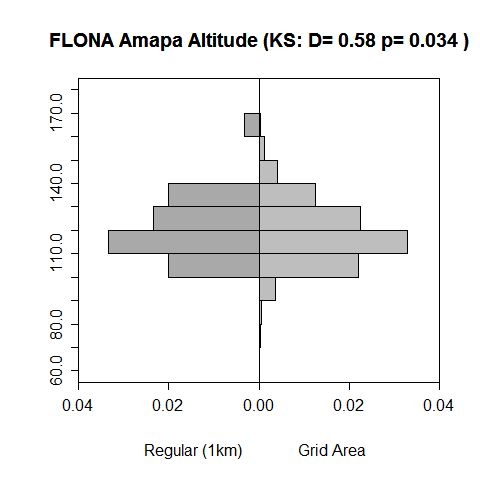 | 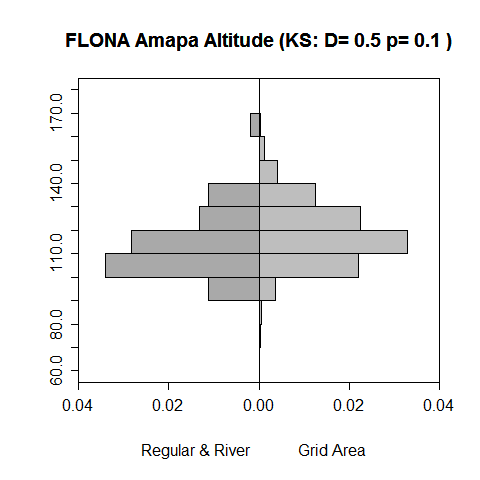 | 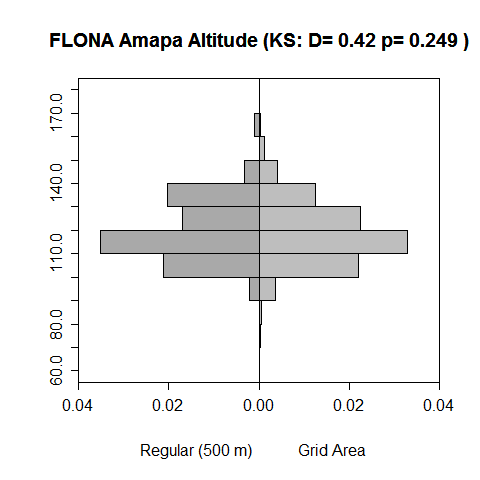 |
| --- | --- | --- |
| 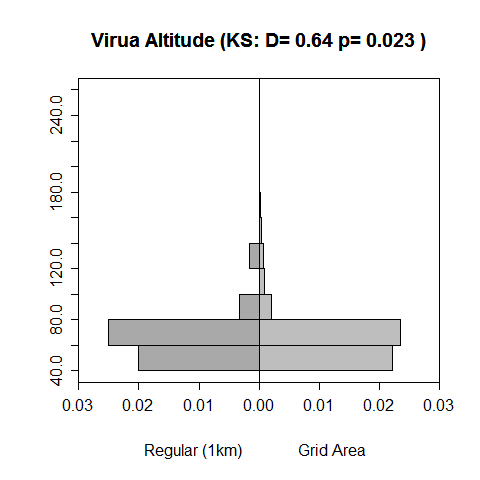 | 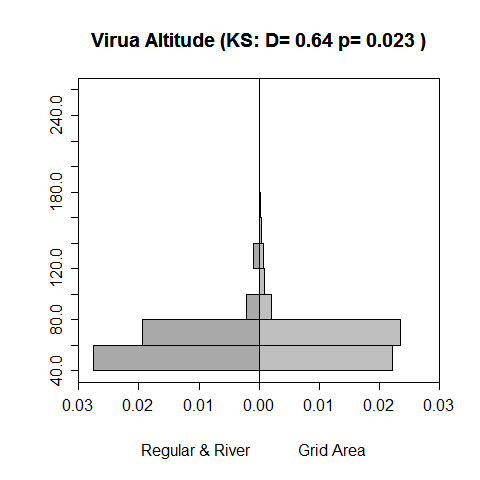 | 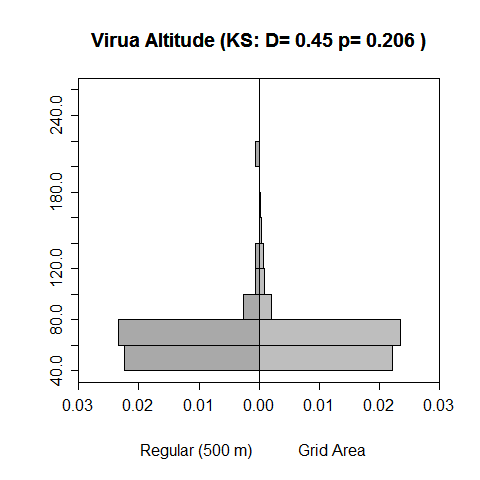 |

Figure S8. Sample “unrepresentativeness”. Sample representativeness across grids that showed sample distributions that were significantly different from the grid altitude values (FLONA Amapá and Virua). Although sample and grid values were statistically different visual examination shows that both sample and grid values have similar patterns with relatively long-tailed distributions. Columns show comparison at different sample sizes (n=30, 49-53, 96).
