# Supplementary material for: Towards Monitoring Biodiversity in Amazonian Forests: How Regular Samples Capture Meso-Scale Altitudinal Variation in 25 km2 Plots
Source: PLoS One. 2014 Aug 29;9(8):e106150. doi: 10.1371/journal.pone.0106150 (PMC4149511; doi:10.1371/journal.pone.0106150)
Supplement: Figure S10 — IDW KS-test conditional inference tree. (DOC) [file pone.0106150.s010.doc]

Figure S10 Interpolation KS-test conditional inference tree.

Figure S10 Conditional inference tree for KS-test *p* values for interpolation representativeness. The tree was used to represent thresholds in the response of KS-test *p* values (comparing the distribution of sample and original SRTM altitude values across 1286 randomly selected areas) according to sample size (“asize”, *n*=4, 8, 16, 30, 60 and 120) and sample heterogeneity (standard deviation “altsd”, ranging from 1 to 50.6). Terminal nodes show a boxplot with median values (bold horizontal bar), 1^st^ and 3^rd^ quartiles (hinges), and approximate 95% confidence intervals (notches) of the group.
