# Supplementary material for: Towards Monitoring Biodiversity in Amazonian Forests: How Regular Samples Capture Meso-Scale Altitudinal Variation in 25 km2 Plots
Source: PLoS One. 2014 Aug 29;9(8):e106150. doi: 10.1371/journal.pone.0106150 (PMC4149511; doi:10.1371/journal.pone.0106150)
Supplement: Figure S12 — IDW error conditional inference tree. (DOC) [file pone.0106150.s012.doc]

Figure S12 IDW error conditional inference tree

Figure S12 Conditional inference tree for the RMSE of IDW interpolations with original SRTM values. Tree was used to represent thresholds in the response of RMSE values (comparing the accuracy of interpolated and original SRTM altitude values across 1286 randomly selected areas) according to sample size (“asize”, *n*=4, 8, 16, 30, 60 and 120) and sample heterogeneity (standard deviation “altsd”, ranging from 1 to 50.6). Terminal nodes show a boxplot with median values (bold horizontal bar), 1^st^ and 3^rd^ quartiles (hinges), and approximate 95% confidence intervals (notches) of the group.
