# Supplementary material for: Towards Monitoring Biodiversity in Amazonian Forests: How Regular Samples Capture Meso-Scale Altitudinal Variation in 25 km2 Plots
Source: PLoS One. 2014 Aug 29;9(8):e106150. doi: 10.1371/journal.pone.0106150 (PMC4149511; doi:10.1371/journal.pone.0106150)
Supplement: Figure S13 — Mapped IDW estimates. (DOC) [file pone.0106150.s013.doc]

Figure S13 Mapped IDW estimates

|  | **Cuniã (SD = 4.8)** |  |
| --- | --- | --- |
|  | | |
|  | **FLONA (SD= 13.5)** |  |
|  | | |
|  | **Ducke (SD = 18.9)** |  |
|  | | |
|  | **Uatuma (SD = 40.2)** |  |
|  | | |

Figure S13 Mapped altitude in 25km^2^ of Amazonian forest. Illustrative example of IDW interpolations obtained within four active research areas (rows). Figure columns present original SRTM altitude values (“ALT SRTM”) and IDW interpolations from different sample sizes, with crosses showing the locations of sample points.
