# Supplementary material for: Towards Monitoring Biodiversity in Amazonian Forests: How Regular Samples Capture Meso-Scale Altitudinal Variation in 25 km2 Plots
Source: PLoS One. 2014 Aug 29;9(8):e106150. doi: 10.1371/journal.pone.0106150 (PMC4149511; doi:10.1371/journal.pone.0106150)
Supplement: Table S1 — Active research areas. (DOC) [file pone.0106150.s014.doc]

Table S1. Active research areas

Summary of Amazonian “active research” study areas. Location and altitude of seven areas distributed across Brazilian Amazonia. Survey areas are separated by approximately 1500 km North – South and 1500 km east - west.

| State | Name | Geographic coordinates^a^ | | ^b^ Alt  mean±SD (range) | # Samples | | Forest Cover Class |
| --- | --- | --- | --- | --- | --- | --- | --- |
|  |  | Longitude | Latitude |  | Regular | River |  |
| Rondônia | Cuniã | -63.47157 | -8.0924903 | 81±4.8(62 – 93) | 30 | 22 | Terra Firme |
| Amazonas | Ducke | -59.94090 | -2.9643581 | 91±18.9(53 – 127) | 30 | 17 | Terra Firme |
| Amapá | FLONA Amapa | -51.63714 | 0.9887931 | 118±13.5(69 – 177) | 30 | 23 | Terra Firme |
| Roraima | Maraca | -61.46453 | 3.3824250 | 119±12.3(78 – 155) | 30 | 19 | Cerrado/Semi-deciduous |
| Amazonas | UFAM | -60.09571 | -2.6406963 | 86±19.3(48 – 125) | 31 | 14 | Terra Firme |
| Amazonas | Uatumã | -59.25121 | -1.8041924 | 149±40.2(58 – 219) | 30 | 18 | Terra Firme |
| Roraima | Virua | -61.02516 | 1.4636897 | 64±19.3(45 – 257) | 30 | 19 | Terra Firme /Flooded Forest |

^a^ Center point of sample area

^b^. Altitude values within sample areas in meters above sea level according to SRTM DEM
